# Clinical characteristics of 1279 patients with hepatitis E in Tianjin

**DOI:** 10.1017/S0950268823001516

**Published:** 2023-09-14

**Authors:** Hang Yang, Jun Wen, Qian Zhang, Chen Chen, Haixia Ma, Lili Zhao, Jia Li

**Affiliations:** 1 Department of Gastroenterology and Hepatology, Tianjin Second People's Hospital, Tianjin, China; 2 Clinical School of the Second People's Hospital, Tianjin Medical University, Tianjin, China

**Keywords:** epidemiology, hepatitis E, liver failure, viral hepatitis vaccines, primary prevention

## Abstract

Hepatitis E virus infection is a major cause of acute hepatitis, typically self-limiting but occasionally leading to liver failure. Understanding disease progression factors could inform prevention strategies. This study aimed to analyse the characteristics of a large cohort of hospitalised hepatitis E patients in Tianjin, China, and explore factors influencing their progression to liver failure. A total of 1279 hospitalised patients with hepatitis E were included in this cross-sectional study in Tianjin, China. Student's *t-*test and the Mann–Whitney *U-*test were used for comparisons. Multiple logistic regression analysis was used to explore the association. Among these 1279 patients, 107 (8.4%) developed liver failure. Patients with diabetes mellitus (DM) (95% confidence interval [*CI*] 1.150–2.887, *p* = 0.011), liver cirrhosis (95% [*CI*] 2.229–7.224, *p* < 0.001), and hepatitis B (95% [*CI*] 1.159–4.512, *p* = 0.017) were more likely to progress to liver failure. Hepatitis E patients with comorbid DM, liver cirrhosis, or hepatitis B virus co-infection have higher risks of developing liver failure. Hepatitis E vaccination may be recommended for these vulnerable patients to curb disease severity.

## Introduction

The hepatitis E virus (HEV) is a long-neglected RNA virus that was discovered in 1983 [1]. As the major common causative agent of acute viral hepatitis in humans worldwide, it is mainly transmitted by faecal–oral route [2–4]. The vast majority of patients are clinically silent, less than five per cent of patients have acute self-limiting hepatitis symptoms, and acute liver failure occurs in very rare cases [5]. About one-third of the world's population have been infected with HEV [6, 7]. HEV has been neglected for a long time. The prevalence status of hepatitis E has been changing, and the epidemiology of HEV remains significant [1].

The seroprevalence of anti-HEV in the general population in China is about 23.46%. In the past 20 years, with the significant improvement of public health conditions in China, the predominant circulating genotypes have gradually switched from genotype 1 to genotype 4, and sporadic cases have become the major form [8]. An HEV vaccine was approved for use in China in 2012, and its uptake is currently limited. [7, 9].

To optimise hepatitis E vaccination strategies, we conducted a study on hepatitis E patients admitted to Tianjin Second People's Hospital, the infectious disease specialty hospital in Tianjin. This hospital admits and treats all hepatitis E patients in Tianjin, including those initially hospitalised elsewhere who are transferred after infectious disease screening reveals hepatitis E infection. By studying this comprehensive cohort of hospitalised hepatitis E patients in Tianjin, we investigated factors associated with liver failure and explored vaccination strategies with the hepatitis E vaccine.

## Methods

### Study population

We reviewed the medical records of hepatitis E patients admitted between January 2010 and May 2023 at Tianjin Second People's Hospital, Tianjin, China. The diagnosis of hepatitis E was made if patients met any of the following criteria: elevated serum alanine aminotransferase (ALT) along with either of the following: serum HEV RNA positive, HEV RNA and anti-HEV IgM positive, HEV RNA and anti-HEV IgG positive, HEV RNA and both anti-HEV IgM and IgG positive, rising anti-HEV IgM and IgG titres, or positive HEV antigen [10]. Among the 1,279 patients included, no cases of chronic HEV infection were identified in this study. Liver failure [11], liver cirrhosis based on a pre-existing diagnosis [12], coronary disease [13], hypertension [14], diabetes mellitus (DM) [15], non-alcoholic fatty liver disease (NAFLD) [16], hepatitis B [17], autoimmune liver disease [18], and alcoholic liver disease (ALD) [19] were diagnosed according to the respective clinical practice guidelines. Steatotic liver disease (SLD) was diagnosed according to the respective consensus [20]. Among them, the diagnostic criteria for acute liver failure are the development of coagulopathy and any degree of encephalopathy in a patient without pre-existing liver disease or cirrhosis over 26 weeks. The total number of hepatitis E cases in Tianjin from 2010 to 2019 was retrieved from the Chinese Center for Disease Control and Prevention.

A total of 1279 hepatitis E patients were included in this cross-sectional analysis. The study protocol was reviewed and approved by the Ethics Committee of Tianjin Second People’s Hospital, in accordance with the ethical principles outlined in the 1975 Declaration of Helsinki (as revised in Brazil, 2013). Informed consent was obtained from each patient before inclusion in the study.

### General characteristics and laboratory test results

The medical records of all 1279 hepatitis E patients were reviewed in this study. Demographic data include gender and age, and laboratory results include blood tests, biochemistry tests, and immunology tests. However, FibroScan data including controlled attenuation parameter (CAP) and liver stiffness measurement (LSM) were collected and analysed. For all 1279 patients, platelet (PLT) count and international normalised ratio (INR) in whole blood were tested using a Sysmex XN-2000 Hematology Analyzer (Sysmex Corporation, Kobe, Japan). Serum ALT, aspartate aminotransferase (AST), albumin (ALB), and total bilirubin (TBil) were tested using a Hitachi 7180 Automatic Biochemical Analyzer (Hitachi Limited, Tokyo, Japan). CAP and LSM were assessed using a FibroScan 502 Touch device (Echosens Limited, Paris, France). Patients underwent routine abdominal ultrasound to measure spleen diameter and thickness across the hilum. Spleen area was defined as the product of diameter and thickness.

### Statistical analysis

The Kolmogorov–Smirnov or Shapiro–Wilk test was used to determine whether continuous variables conformed to a normal distribution. Variables conforming to a normal distribution were expressed as mean and standard deviation, while non-normal variables were expressed as median and quartile range, and categorical variables were expressed as frequency and percentage. One thousand two hundred and seventy-nine hepatitis E patients were enrolled in this cross-sectional study. Student's *t*-test or the Mann–Whitney *U*-test was used to compare groups. Multiple logistic regression analysis was used to explore the association between clinical variables and liver failure. *p* < 0.050 was considered statistically significant. Statistical analyses were performed using SPSS version 27 and R (tidyverse, caret, and epiR packages).

## Results

### General characteristics of patients with hepatitis E

**Figure 1.**
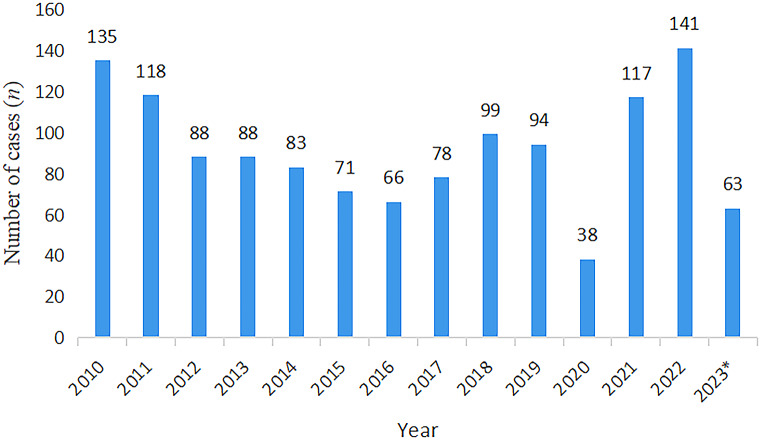
Time distribution of hepatitis E patients*: Number of patients from January to May, 2023.

**Figure 2.**
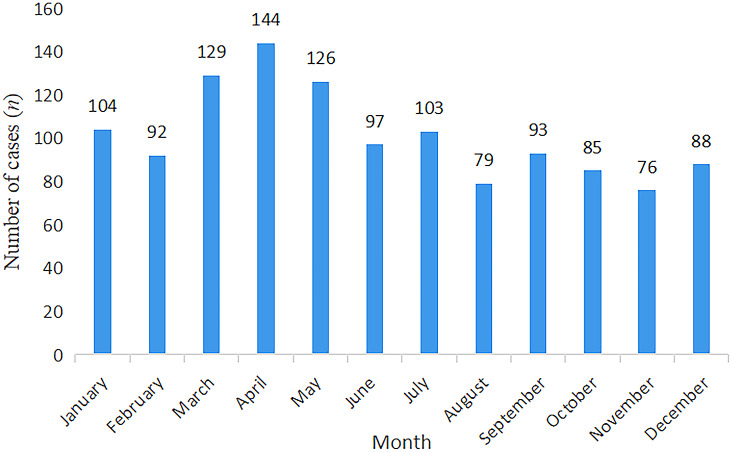
Time distribution of hepatitis E patients

Of the 1,279 hepatitis E patients in this study, 964 (75.4%) were male. In addition to hepatitis E, 29 patients had cancer, 17 had autoimmune hepatitis (AIH), nine had primary biliary cholangitis (PBC), six had hepatitis C, and one had hereditary and metabolic liver disorders (Supplementary Table S1). The annual (Figure 1) and monthly (Figure 2) distribution of hepatitis E cases is shown in the figures (Supplementary Table S2). The incidence of hepatitis E was highest in April. Among hepatitis E cases in Tianjin from 2010 to 2019, 72.2% required hospitalisation. HIV testing was performed for all patients, and no infections were identified. Of the six patients with hepatitis C virus infection, three had an acute infection, two had a chronic active infection, and one had a past infection.

### General characteristics of patients with hepatitis E caused by liver failure

Of the hepatitis E patients in this study, 107 (8.4%) developed liver failure. Patients with liver failure were older (*p* = 0.042), had lower PLT (*p* < 0.001), higher INR (*p* < 0.001), higher serum ALT levels (*p* < 0.001), higher AST levels (*p* < 0.001), lower ALB levels (*p* < 0.001), higher TBil levels (*p* < 0.001), higher LSM (*p* < 0.001), higher rates of DM (*p* = 0.011), higher rates of liver cirrhosis (*p* < 0.001), larger spleen sizes (*p* < 0.001), and higher rates of hepatitis B (*p* = 0.019). There were no significant differences in gender, CAP, coronary disease, hypertension, NAFLD, ALD, or pregnancy between the two groups (*p* ≥ 0.050) (Table 1).

**Table 1.** General characteristics of patients with hepatitis E by hepatic failure

Variables	Hepatic failure patients (*n* = 107)	Other patients (*n* = 1172)	*p*
Male	89 (83.2)	874 (74.6)	0.060
Age (years)	60 (51,66)	57 (48,64)	0.042
PLT (U/L)	130.00 (90.00,173.5)	190.00 (146.00,244.00)	<0.001
INR	1.60 (1.29,1.96)	1.00 (0.92,1.12)	<0.001
ALT (U/L)	978.00 (392.00,1934.00)	642.50 (259.50,1279.75)	<0.001
AST (U/L)	798.50 (253.50,1320.50)	232.50 (78.25,671.25)	<0.001
ALB (U/L)	32.00 (29.00,35.00)	36.00 (32.00,39.00)	<0.001
TBil (μmol/L)	207.00 (146.50,307.50)	95.00 (38.00,178.00)	<0.001
CAP (dB/m)	196.00 (176.00,237.00)	219.00 (188.00,250.50)	0.096
LSM (kPa)	18.00 (13.00,33.50)	10.00 (8.00,14.00)	<0.001
Coronary disease	8 (7.5)	84 (7.2)	1.000
Hypertension	40 (37.4)	359 (30.6)	0.157
DM	32 (30.0)	225 (19.2)	0.011
Liver cirrhosis	17 (17.7)	57 (5.1)	<0.001
Spleen area	4196.00 (3564.00,5581.50)	3710.00 (3036.00,4488.00)	<0.001
NAFLD	38 (35.5)	345 (29.4)	0.225
Hepatitis B	12 (11.2)	63 (5.4)	0.019
ALD	24 (22.4)	200 (17.1)	0.183
Pregnancy	1 (0.9)	11 (0.9)	1.000

*Note:* Data are expressed as mean ± standard deviation, median (quartile 25, quartile 75), or number (proportion).

*Abbreviations:* PLT, platelet; INR, international normalised ratio; ALT, alanine aminotransferase; AST, aspartate aminotransferase; ALB, albumin; TBil, total bilirubin; CAP, controlled attenuation parameter; LSM, liver stiffness measurement; DM, diabetes mellitus; NAFLD, non-alcoholic fatty liver disease; ALD, alcoholic liver disease.

### Addictive interaction between DM and SLD for liver failure risk

Table 2 shows the additive interaction between DM and SLD on the risk of liver failure in patients with hepatitis E. DM increased the risk of liver failure (*OR*: 1.822, 95% *CI*: 1.150–2.887), while SLD did not (*OR*: 1.426, 95% *CI*: 0.945–2.152). There was no evidence of an additive interaction between DM and SLD on the risk of liver failure in patients with hepatitis E.

**Table 2.** Interactive effects between DM and SLD for LF risk

	*OR* (95% *CI*)	*p*
DM versus non-DM	1.822 (1.150, 2.887)	0.011
SLD versus non-SLD	1.426 (0.945, 2.152)	0.091
**Additive interaction model of DM and SLD**		
Non-DM and non-SLD	Reference	
Non-DM and SLD	1.311 (0.862, 1.994)	0.206
DM and non-SLD	Unavailable	
DM and SLD	1.245 (0.695, 2.232)	0.462
*RERI*	0.139 (−0.363, 0.640)	
*APAB*	0.198 (−0.306, 0.702)	
*S*	0.684 (0.683, 6.857)	

*Abbreviations:* DM, diabetes mellitus; SLD, steatotic liver disease.

### Analysis of risk factors associated with liver failure in patients with hepatitis E

Binary logistic regression analysis was used to explore the association between clinical variables and liver failure in patients with hepatitis E. Patients progressing towards liver failure had higher rates of DM (*p* = 0.011), liver cirrhosis (*p* < 0.001), and hepatitis B (*p* = 0.017) (Table 3). After multivariate logistic regression analysis, liver cirrhosis and hepatitis B were found to be independent risk factors for predicting liver failure in hepatitis E patients, with odds ratios (*OR*s) (95% confidence intervals [*CI*s]) of 3.862 (2.136, 6.980) and 2.286 (1.159, 4.512), respectively.

**Table 3.** Univariate and multivariate analysis of risk factors associated with liver failure

Variables	Univariate analysis *OR* (95% *CI*)	*p*	Multivariate analysis *OR* (95% *CI*)	*p*
Gender	1.699 (0.977, 2.954)	0.061		
Age	1.015 (0.998, 1.033)	0.085		
Coronary disease	0.992 (0.467, 2.109)	0.984		
Hypertension	1.359 (0.882, 2.094)	0.164		
DM	1.822 (1.150, 2.887)	0.011		
Liver cirrhosis	4.013 (2.229, 7.224)	<0.001	3.862 (2.136, 6.980)	<0.001
NAFLD	1.510 (0.988, 2.308)	0.057		
CAP	0.991 (0.981, 1.002)	0.098		
Hepatitis B	2.286 (1.159, 4.512)	0.017	2.286 (1.159, 4.512)	0.037
ALD	1.058 (0.652, 1.717)	0.818		
Pregnancy	1.061 (0.136, 8.308)	0.955		

*Abbreviations:* DM, diabetes mellitus; NAFLD, non-alcoholic fatty liver disease; CAP, controlled attenuation parameter; ALD, alcoholic liver disease.

### General characteristics of patients by mortality

Seventeen (15.9%) hepatitis E patients died in this study. Patients who died had a higher MELD-Na score (*p =* 0.033) and a higher INR (*p =* 0.001). There were no significant differences in gender, age, types of liver failure, PLT, ALT, AST, ALB, TBil, liver cirrhosis, hepatitis B, DM, SLD, and pregnancy between these two groups (*p* ≥ 0.050) (Table 4).

**Table 4.** General characteristics of hepatic failure patients with hepatitis E

Variables	Dead (*n* = 17)	Alive (*n* = 90)	*p*
Male	13 (76.5)	76 (84.4)	0.480
Age (years)	57.5 (52,63.75)	60.5 (50.25,66)	0.576
MELD-Na	24.1 (11.25,35.18)	15.29 (11.03,18.93)	0.033
Liver failure			0.468
Acute	4 (23.6)	17 (18.9)	
Subacute	8 (47.0)	55 (61.1)	
Acute on chronic	5 (29.4)	18(20.0)	
INR	2 (1.76,3.8)	1.46 (1.22,1.85)	0.001
PLT (U/L)	133 (97.25,226.5)	125 (88.5,171)	0.390
ALT (U/L)	1538.94 ± 1166.65	1332.18 ± 1190.50	0.528
AST (U/L)	1030 (470.5,1708.75)	732.5 (202.75,1245)	0.156
ALB (U/L)	32 (28,34.75)	32 (29,35.5)	0.371
TBil (μmol/L)	252.06 ± 118.18	239.32 ± 122.97	0.705
Liver cirrhosis	3 (17.7)	14 (15.6)	1.000
Hepatitis B	1 (5.9)	11 (12.2)	0.686
DM	2 (11.8)	27 (30.0)	0.147
SLD	7 (41.2)	49 (54.4)	0.428
Pregnancy	1 (5.9)	0	0.159

*Note:* Data are expressed as mean ± standard deviation, median (quartile 25, quartile 75), or number (proportion).

*Abbreviations:* MELD-Na, model for end-stage liver disease-Na; INR, international normalised ratio; PLT, platelet; ALT, alanine aminotransferase; AST, aspartate aminotransferase; ALB, albumin; TBil, total bilirubin; DM, diabetes mellitus; SLD, steatotic liver disease.

## Discussion

Hepatitis E is caused by infection with the hepatitis E virus (HEV). HEV is one of the leading causes of acute viral hepatitis worldwide and belongs to the Hepeviridae family. Its prevalence varies in different regions [1–3, 21]. HEV was first characterised in detail in 1983 during an outbreak of non-A, non-B, and non-C hepatitis among Soviet soldiers on a military mission in Afghanistan. Russian virologist Balayan visualised the virus using electron microscopy while examining one of their own stool samples after ingesting a pooled faecal extract from infected soldiers [22]. Severe hepatitis caused by HEV includes acute liver failure and chronic liver failure. It can cause acute decompensation of chronic liver disease, leading to liver failure and even death. In this study, the overall mortality rate for hospitalised hepatitis E patients in Tianjin over the past 14 years was 1.6%.

Patients could be infected with HEV throughout the year, but the incidence rate was highest in April. This seasonal peak might be related to weather conditions in spring that are more favourable for virus multiplication and spread. However, further research is needed to validate this trend. However, our study focused on hospitalised patients, so the data on HEV hospitalisations in 2020 might have been affected primarily by COVID-19 restrictions and lockdowns rather than changes in actual incidence. Another noticeable feature of HEV in our study was that male patients significantly outnumbered female patients, which is consistent with findings from other reports [23]. The reasons for the higher incidence of acute hepatitis E in men compared with women remain unclear.

The results of this study found that hepatitis E combined with liver cirrhosis or hepatitis B is more likely to progress to liver failure. This study also found that patients with hepatitis E who progressed to liver failure had lower platelet counts and smaller spleen sizes, which may also be due to these patients having liver cirrhosis [23]. Zhao et al. [24] investigated the specific role of HEV superinfection in the long-term outcome of hepatitis B virus (HBV) patients with liver cirrhosis and found that co-infection was associated with higher mortality. Many studies have reported that HEV infection could accelerate disease progression in patients with underlying chronic liver disease and increase morbidity and mortality in these patients [25–28]. These findings are consistent with the results of this study. Furthermore, similar to HEV, hepatitis A virus (HAV) superinfections in patients with underlying hepatitis B have also been associated with increased liver-related mortality. During a large hepatitis A outbreak in Shanghai, China, in 1988 related to the consumption of raw clams, increased deaths were observed in patients with hepatitis B surface antigen positivity [29]. The risks of acute liver failure and mortality associated with HAV or HEV co-infection highlight the need for vaccination against both viruses in chronic hepatitis B patients. In addition to chronic liver disease, this study also examined the impact of chronic diseases (coronary heart disease, hypertension, and DM) on the progression of hepatitis E patients to liver failure. It found that patients with DM rather than SLD were more likely to progress to liver failure, which is consistent with the findings of Tarantino et al. [30]. This study did not find evidence of an additive interaction between DM and SLD on the risk of liver failure in hepatitis E patients.

Pregnant women may exhibit more severe symptoms upon HEV infection, and the infection can be fatal in this population [31–34]. This study found that pregnant women with hepatitis E in Tianjin did not have an additional risk of progressing to liver failure. This is consistent with many other studies, suggesting that progression to liver failure in pregnant women may be related to many local factors such as HEV genotypes and living conditions, rather than an independent risk factor [35–38].

Some patients with hepatitis E who progressed to liver failure ultimately die. These patients tend to have a higher prevalence of liver cirrhosis, indicating more severe and advanced liver disease. Our study found that the MELD-Na score and INR were useful for predicting mortality from liver failure, consistent with the findings of Wallace et al. [39]. Monitoring these indicators could help identify patients at greatest risk so that healthcare resources can be efficiently allocated. More intensive monitoring and treatment may help improve outcomes, though liver transplantation ultimately provides the only definitive treatment for those experiencing liver failure.

This large, long-term study included all hospitalised hepatitis E patients in Tianjin over the past 14 years, providing useful descriptive data. However, it had limitations. The lack of a DM-only group potentially biased the assessment of the DM-SLD interaction on liver failure risk. Further research should stratify DM subgroups to clarify this interaction. Additionally, the cross-sectional design prevented determining causality. Therefore, prospective studies are warranted to confirm findings and establish causation. Other limitations include a lack of paediatric data and genotype data. However, it captured a substantial adult sample over a prolonged period. Future directions should involve prospective research on diverse populations, including children, along with more complete clinical and genotype data to elucidate mechanisms.

Hepatitis E vaccination could reduce the risk of hepatitis E in recipients. It has already been used in China and Pakistan for individuals travelling abroad to areas with a high risk of hepatitis E [40]. While this provides protection for travellers, vulnerable groups within populations also need to be identified and immunised. Patients with DM, liver cirrhosis, and hepatitis B who become infected with HEV are more prone to progressing to liver failure. To prevent this, early identification of these at-risk groups is important. Furthermore, the WHO has launched an initiative to eliminate viral hepatitis by 2030. To achieve this goal, those with DM, liver cirrhosis, and hepatitis B should receive the hepatitis E vaccine. Immunising these vulnerable groups could help reduce not only the health threat but also the financial burden of the disease.

## Supporting information

Yang et al. supplementary material Yang et al. supplementary material

## Data Availability

The data used and analysed during this study are available from the corresponding author upon reasonable request.

## Supplementary material

For supplementary material accompanying this paper visit http://doi.org/10.1017/S0950268823001516. click here to view supplementary material
