# Supplementary material for: Clinical characteristics of 1279 patients with hepatitis E in Tianjin
Source: Epidemiol Infect. 2023 Sep 14;151:e157. doi: 10.1017/S0950268823001516 (PMC10548536; doi:10.1017/S0950268823001516)
Supplement: Yang et al. supplementary material [file S0950268823001516sup001.doc]

*Epidemiology and Infection*

## Clinical characteristics of 1279 patients with hepatitis E in Tianjin

## Hang Yang, Jun Wen, Qian Zhang,Weiming Luo, Chen Chen, Haixia Ma, Lili Zhao, Jia Li

Supplementary Material

**Supplementary Table S1.** General characteristics of patients with hepatitis E

| Variables | Value |
| --- | --- |
| Male | 964 (75.4) |
| Age (years) | 57 (48,64) |
| Liver cirrhosis | 74 (5.8) |
| Coronary disease | 92 (7.2) |
| Hypertension | 399 (31.2) |
| DM | 257 (20.1) |
| NAFLD | 383 (29.9) |
| Hepatitis B | 75 (5.9) |
| Autoimmune liver disease | 26 (2.0) |
| ALD | 224 (17.5) |

Data are expressed as median (quartile 25,quartile 75) or number (proportion)

DM, diabetes mellitus; NAFLD, non-alcoholic fatty liver disease; ALD, alcoholic liver disease

**Supplementary Table S2.** The annual and monthly distribution of hepatitis E cases

| Year | Month | Cases |
| --- | --- | --- |
| 2010 | January | 15 |
| 2011 | January | 16 |
| 2012 | January | 5 |
| 2013 | January | 7 |
| 2014 | January | 9 |
| 2015 | January | 9 |
| 2016 | January | 6 |
| 2017 | January | 5 |
| 2018 | January | 6 |
| 2019 | January | 7 |
| 2020 | January | 8 |
| 2021 | January | 1 |
| 2022 | January | 10 |
| 2010 | February | 9 |
| 2011 | February | 8 |
| 2012 | February | 7 |
| 2013 | February | 9 |
| 2014 | February | 4 |
| 2015 | February | 6 |
| 2016 | February | 7 |
| 2017 | February | 5 |
| 2018 | February | 10 |
| 2019 | February | 8 |
| 2020 | February | 5 |
| 2021 | February | 5 |
| 2022 | February | 9 |
| 2010 | March | 14 |
| 2011 | March | 10 |
| 2012 | March | 10 |
| 2013 | March | 15 |
| 2014 | March | 5 |
| 2015 | March | 8 |
| 2016 | March | 7 |
| 2017 | March | 7 |
| 2018 | March | 10 |
| 2019 | March | 5 |
| 2020 | March | 3 |
| 2021 | March | 17 |
| 2022 | March | 18 |
| 2010 | April | 24 |
| 2011 | April | 18 |
| 2012 | April | 8 |
| 2013 | April | 7 |
| 2014 | April | 11 |
| 2015 | April | 5 |
| 2016 | April | 11 |
| 2017 | April | 7 |
| 2018 | April | 13 |
| 2019 | April | 12 |
| 2020 | April | 4 |
| 2021 | April | 8 |
| 2022 | April | 16 |
| 2010 | May | 12 |
| 2011 | May | 14 |
| 2012 | May | 14 |
| 2013 | May | 12 |
| 2014 | May | 10 |
| 2015 | May | 5 |
| 2016 | May | 6 |
| 2017 | May | 8 |
| 2018 | May | 6 |
| 2019 | May | 6 |
| 2020 | May | 3 |
| 2021 | May | 19 |
| 2022 | May | 11 |
| 2010 | June | 8 |
| 2011 | June | 10 |
| 2012 | June | 6 |
| 2013 | June | 4 |
| 2014 | June | 7 |
| 2015 | June | 9 |
| 2016 | June | 9 |
| 2017 | June | 12 |
| 2018 | June | 6 |
| 2019 | June | 4 |
| 2020 | June | 0 |
| 2021 | June | 8 |
| 2022 | June | 14 |
| 2010 | July | 12 |
| 2011 | July | 14 |
| 2012 | July | 12 |
| 2013 | July | 7 |
| 2014 | July | 6 |
| 2015 | July | 5 |
| 2016 | July | 3 |
| 2017 | July | 6 |
| 2018 | July | 7 |
| 2019 | July | 9 |
| 2020 | July | 1 |
| 2021 | July | 13 |
| 2022 | July | 8 |
| 2010 | August | 13 |
| 2011 | August | 5 |
| 2012 | August | 4 |
| 2013 | August | 6 |
| 2014 | August | 3 |
| 2015 | August | 5 |
| 2016 | August | 3 |
| 2017 | August | 3 |
| 2018 | August | 9 |
| 2019 | August | 12 |
| 2020 | August | 5 |
| 2021 | August | 5 |
| 2022 | August | 6 |
| 2010 | September | 6 |
| 2011 | September | 9 |
| 2012 | September | 5 |
| 2013 | September | 4 |
| 2014 | September | 7 |
| 2015 | September | 3 |
| 2016 | September | 4 |
| 2017 | September | 8 |
| 2018 | September | 8 |
| 2019 | September | 13 |
| 2020 | September | 4 |
| 2021 | September | 9 |
| 2022 | September | 13 |
| 2010 | October | 10 |
| 2011 | October | 6 |
| 2012 | October | 6 |
| 2013 | October | 6 |
| 2014 | October | 6 |
| 2015 | October | 4 |
| 2016 | October | 5 |
| 2017 | October | 5 |
| 2018 | October | 11 |
| 2019 | October | 6 |
| 2020 | October | 2 |
| 2021 | October | 12 |
| 2022 | October | 6 |
| 2010 | November | 9 |
| 2011 | November | 5 |
| 2012 | November | 2 |
| 2013 | November | 4 |
| 2014 | November | 9 |
| 2015 | November | 7 |
| 2016 | November | 3 |
| 2017 | November | 6 |
| 2018 | November | 5 |
| 2019 | November | 3 |
| 2020 | November | 1 |
| 2021 | November | 11 |
| 2022 | November | 11 |
| 2010 | December | 3 |
| 2011 | December | 3 |
| 2012 | December | 9 |
| 2013 | December | 7 |
| 2014 | December | 6 |
| 2015 | December | 5 |
| 2016 | December | 2 |
| 2017 | December | 6 |
| 2018 | December | 8 |
| 2019 | December | 9 |
| 2020 | December | 2 |
| 2021 | December | 9 |
| 2022 | December | 19 |
